# Evaluation of Parkinson’s disease early diagnosis using single-channel EEG features and auditory cognitive assessment

**DOI:** 10.3389/fneur.2023.1273458

**Published:** 2023-12-19

**Authors:** Lior Molcho, Neta B. Maimon, Neomi Hezi, Talya Zeimer, Nathan Intrator, Tanya Gurevich

**Affiliations:** ^1^Neurosteer Inc., New York, NY, United States; ^2^Department of Musicology, The Hebrew University of Jerusalem, Jerusalem, Israel; ^3^Movement Disorders Unit, Neurological Institute, Tel Aviv Sourasky Medical Center, Tel Aviv, Israel; ^4^Blavatnik School of Computer Science, Tel Aviv University, Tel Aviv, Israel; ^5^Sagol School of Neuroscience, Tel Aviv University, Tel Aviv, Israel; ^6^Faculty of Medicine, Tel Aviv University, Tel Aviv, Israel

**Keywords:** electroencephalography, biomarkers, Parkinson’s disease, F-DOPA PET scan, dopamine, cognition, cognitive assessment, machine learning

## Abstract

**Background:**

Parkinson’s disease (PD) often presents with subtle early signs, making diagnosis difficult. F-DOPA PET imaging provides a reliable measure of dopaminergic function and is a primary tool for early PD diagnosis. This study aims to evaluate the ability of machine-learning (ML) extracted EEG features to predict F-DOPA results and distinguish between PD and non-PD patients. These features, extracted using a single-channel EEG during an auditory cognitive assessment, include EEG feature A0 associated with cognitive load in healthy subjects, and EEG feature L1 associated with cognitive task differentiation.

**Methods:**

Participants in this study are comprised of cognitively healthy patients who had undergone an F-DOPA PET scan as a part of their standard care (*n* = 32), and cognitively healthy controls (*n* = 20). EEG data collected using the Neurosteer system during an auditory cognitive task, was decomposed using wavelet-packet analysis and machine learning methods for feature extraction. These features were used in a connectivity analysis that was applied in a similar manner to fMRI connectivity. A preliminary model that relies on the features and their connectivity was used to predict initially unrevealed F-DOPA test results. Then, generalized linear mixed models (LMM) were used to discern between PD and non-PD subjects based on EEG variables.

**Results:**

The prediction model correctly classified patients with unrevealed scores as positive F-DOPA. EEG feature A0 and the Delta band revealed distinct activity patterns separating between study groups, with controls displaying higher activity than PD patients. In controls, EEG feature L1 showed variations between resting state and high-cognitive load, an effect lacking in PD patients.

**Conclusion:**

Our findings exhibit the potential of single-channel EEG technology in combination with an auditory cognitive assessment to distinguish positive from negative F-DOPA PET scores. This approach shows promise for early PD diagnosis. Additional studies are needed to further verify the utility of this tool as a potential biomarker for PD.

## 1 Introduction

Diagnosing Parkinson’s Disease (PD) at early stages may be challenging as clinical signs can be subtle, inconclusive, and require differentiation from other disorders. To validate their diagnosis in the early stages, clinicians utilize objective biomarkers of dopaminergic degeneration. Positron emission tomography (PET) scans with [18F]-6-fluoro-L-3,4-dihydroxyphenylalanine (F-DOPA) is an established FDA-approved technique for PD diagnosis (1). Despite the effectiveness of PET scans in diagnosing PD, several challenges limit their broader use: they are large and costly, involve 30 min of radiation exposure using radioactive tracers (2), requires trained personnel, and requires the patient to lay still, which is particularly challenging for seniors with tremor symptoms. Moreover, these scans often are not covered by health insurance, adding financial burdens (3). As the pursuit of disease-modifying treatments for PD intensifies, there is a growing demand for new, cost-effective biomarkers that facilitate early diagnosis and are more accessible in clinical settings (4).

Electroencephalographic (EEG) signals have been extensively studied for over a century and are generally used to investigate cortical and subcortical functionality (5). EEG offers a low-cost and non-invasive approach, directly measuring neural activity, which can be analyzed in various dimensions, including time, space, frequency, power, and phase, reflecting specific neurophysiological mechanisms (6). Advancements in ML and signal processing techniques, such as multi-taper analysis (7, 8) have significantly contributed to extracting useful information from raw EEG signals (9). Novel techniques can exploit the vast amount of information on time-frequency processes in a single recording (10, 11).

Since the loss of dopaminergic neurons affects multiple brain networks, EEG could serve as a research tool in PD (12). Quantitative EEG (qEEG) provides a reliable and widely available measurement that could yield biomarkers for disease severity in PD patients (13). Generally, the incidence of EEG abnormalities in PD patients is higher than in healthy elderly individuals, with the most common alteration being generalized slowing of the EEG (14, 15). Some research is available regarding PD diagnosis; for instance, coherence function (*CF*) has been hypothesized to be a relevant tool for detecting early PD signs (16). *CF* is related to cortical dynamic imbalances and measures linear dependence through the frequency domain between a pair of electrodes placed on the scalp (17). Coherence can detect changes in functional and effective cortical interconnections that occur in the initial onset of PD (18). Indeed, previous studies have reported that non-linear analysis of EEG signals, particularly machine learning (ML) methods, can extract features that could potentially serve as PD biomarkers (19–25). A recent study published results discriminating early-stage PD from healthy brain function using multi-EEG event-related potentials (ERPs) combined with brain network analytics and ML tools while participants performed an auditory cognitive assessment (26).

In this pilot study, we evaluated the ability of an easy-to-use single-channel EEG system (by Neurosteer®) combined with an auditory cognitive assessment to detect electrical activity changes associated with PD. Past research indicates that capturing EEG data during active participation in cognitive and auditory tasks can reveal unique features, potentially enhancing the discrimination power of different brain states (27). Of particular interest are two biomarkers: features A0 and L1. EEG feature A0, previously identified as a classifier distinguishing cognitive load from rest in healthy subjects, has been recognized as a potent predictor of cognitive decline in individuals with mild-to-moderate impairment (28). Whereas EEG feature L1 acted as an LDA classifier, which was developed and validated on datasets involving participants undergoing an n-back working memory task (29). Both features were selected due to their demonstrated robustness and consistent capability to differentiate between cognitive states and task levels (29, 30). It is essential to note that the participants holding a valid F-DOPA test included in this study had no prior diagnosis of cognitive decline and did not report any neuro-cognitive symptoms. We utilized an auditory assessment with musical stimuli that has been previously employed to distinguish between cognitive decline and healthy senior participants (28, 31). The objective of the present study was to assess the capability of these features extracted from a single-channel EEG during an auditory cognitive assessment, to differentiate between positive and negative F-DOPA PET results in order to potentially discriminate between PD and non-PD populations.

## 2 Materials and methods

### 2.1 Participants

This study included 32 participants (15 females) with a mean age of 64.5 (SD = 11.73), all holding a valid F-DOPA PET scan results obtained as part of their standard care due to clinical symptoms suspicious of early PD. Additionally, 20 age-matched, cognitively healthy individuals (7 females) with a mean age of 66.61 (SD = 5.33) served as controls. The entire cohort of 52 participants underwent assessment at rest, and then an auditory cognitive assessment, while their brain activity was recorded using a single-channel electroencephalogram (EEG) by Neurosteer. Informed consent was obtained from each participant before their involvement in the study.

#### 2.1.1 Participants with F-DOPA PET results

Participants with F-DOPA PET results were recruited from the Movement Disorders Unit at Tel Aviv Sourasky Medical Center if they had an MMSE score higher than 24 and could hear, read, and understand instructions for the Informed Consent Form (ICF) discussion as well as for the auditory assessment tasks. Individuals with compromised scalp or skull integrity, facial or forehead skin irritation, hearing loss, cognitive decline and a history of severe drug abuse were excluded from the study.

Ethical approval for data collection was obtained from the Ethics Committee (EC) of Tel Aviv Sourasky Medical Center (Ichilov) on June 07, 2021. Israeli Ministry of Health (MOH) registry number: MOH_2021-06-02_010019.

#### 2.1.2 Healthy participants

For statistical power reasons, since most patients recruited to the study had a positive F-DOPA result, we did an additional analysis with a group of healthy age-matched controls. These patients’ data was taken from another study (NIH registry number: NCT04683835). Out of 80 patients recruited from an orthopedic rehabilitation center, 40 had an MMSE score between 28 and 30. Out of this group, we randomly selected 20 participants to compose the healthy group for the purpose of the current study. Participants were selected randomly to create an age-matched control group (random selection was done using the pandas DataFrame.sample function). The resulting control group was comprised of 7 females and 13 males to match the existing gender distribution among the patient already included in the analysis. The mean age was 66.61 (5.33), and the mean MMSE score was 29.09 (0.78). The full participants selection code is provided in Supplementary materials C.

#### 2.1.3 Study groups

**Figure 1:**
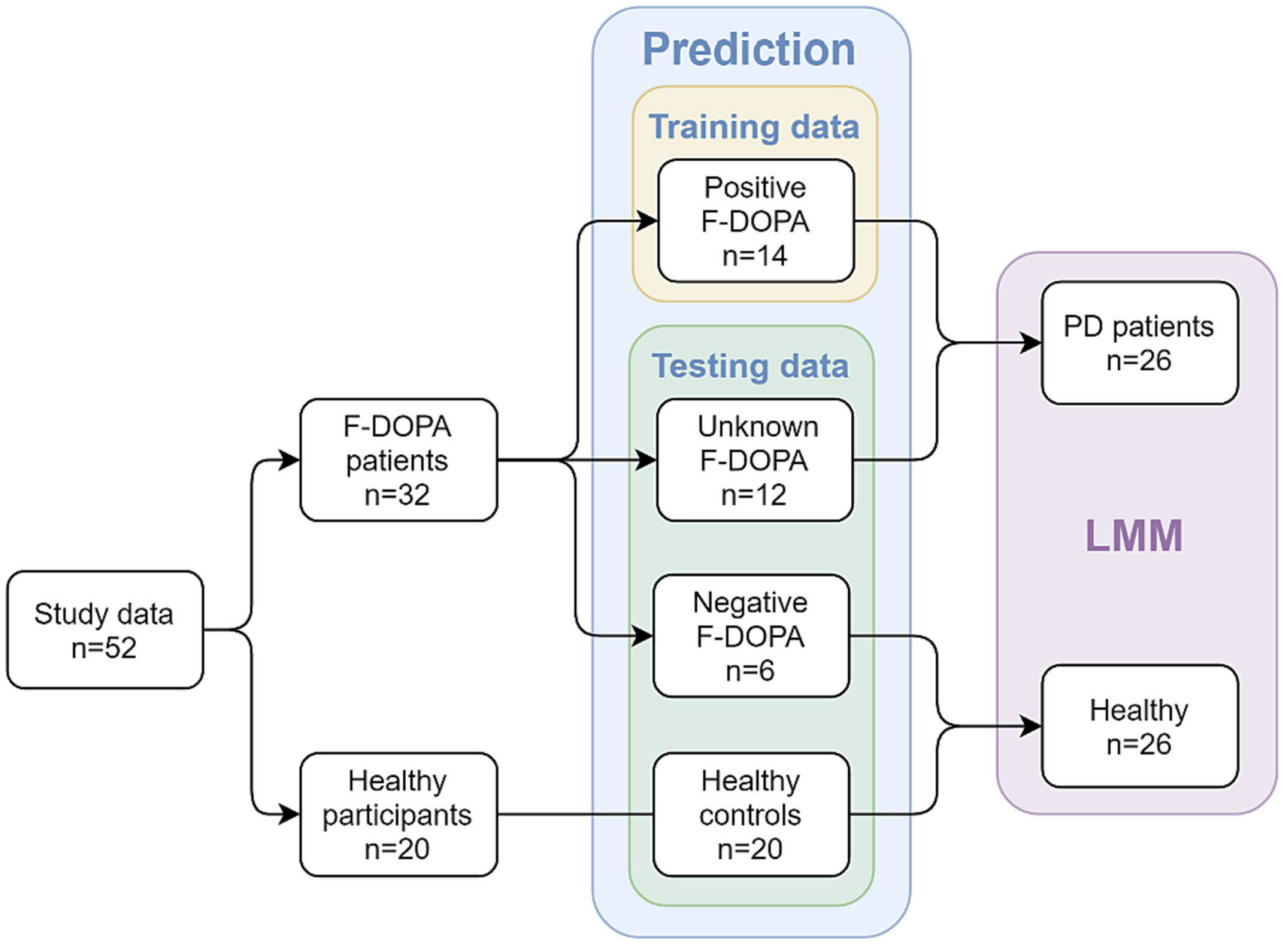
Study design and groups at each analysis stage. The study included patients with valid F-DOPA results and age-matched healthy participants as controls. In the first part of the analysis, a prediction model was used; patients with an initial positive F-DOPA score were included in the model’s training data, while patients with an initial negative F-DOPA score, patients with unrevealed test scores, and healthy controls made up the testing data. In the second part of the analysis (after the results were revealed), a Linear Mixed Model (LMM) was utilized to compare between two combined groups: the PD-patients group (*n* = 26, patients with positive F-DOPA results) and the healthy group (*n* = 26, patients with negative F-DOPA results along with healthy controls).

The 52 study participants were initially divided into four groups based on their F-DOPA results (see Figure 1): participants with a positive F-DOPA result (*n* = 14); participants with a negative F-DOPA result (*n* = 6); participants whose label was initially unrevealed in the ‘unknown’ group (*n* = 12); and healthy age-matched controls (*n* = 20). These groups were used in building and testing the prediction model.

For the second part of the analysis using Linear mixed Models (LMM), the labels were revealed, and participants were added to the relevant groups: the healthy age-matched controls were combined with the negative group to form the ‘healthy’ group (*n* = 26), which was compared to the ‘PD patients’ group consisting of patients with a positive F-DOPA result (*n* = 26, see Healthy participants section above).

To confirm that the groups were balanced in terms of age, gender, and MMSE scores, we analyzed the average ages of each group collectively and for males and females individually. Moreover, we contrasted the age and MMSE scores within each group for both genders. These analyses were performed using the Welch Two Sample *t*-test.

### 2.2 EEG device

EEG measurements were executed utilizing the Recorder (Neurosteer EEG recorder). An FDA cleared adhesive with three electrodes was applied to the subject’s forehead, using a dry gel to enhance signal quality. The non-intrusive electrodes were located at the prefrontal areas, producing a single-EEG channel derived from the difference between Fp1 and Fp2 and a ground electrode at Fpz, based on the international 10/20 electrode positioning. The signal range is ±25 mV (background noise <30nVrms). The electrode contact impedances were kept under 12 kΩ, as determined by a handheld impedance device (EZM4A, Grass Instrument Co., USA). The data was acquired in a continuous mode and subsampled to 500 samples per second.

During the data collection, a proficient research member oversaw each subject to reduce potential muscle interference. Subjects received guidance to refrain from making facial gestures during the session, and the supervising member would notify them if noticeable muscle or eye movements were detected. Notably, the differential signal processing and superior common-mode rejection ratio (CMRR) contribute to minimizing motion disturbances and electrical interference (32).

### 2.3 Procedure

#### 2.3.1 EEG recording and auditory assessment protocol

The session was conducted in a quiet, well-lit room. A research team member prepared the sanitized Neurosteer EEG recording equipment (including the disposable electrode patch, sensor, EEG monitor, and clicker) for deployment. Recording commenced once the electrode patch was attached to the participant’s forehead. Throughout the evaluation, participants remained seated and received guidance through a speaker linked to the EEG monitor. The entire session generally lasted 30 min. The cognitive assessment battery included a musical detection task (with two difficulty levels), a musical n-back task (with two difficulty levels) and a resting-state task. The research assistant provided initial instructions to participants, ensuring minimal additional directives to avert any bias. A brief segment of baseline activity was captured for every participant to guarantee accurate evaluation.

##### 2.3.1.1 Cognitive tasks

**Figure 2:**
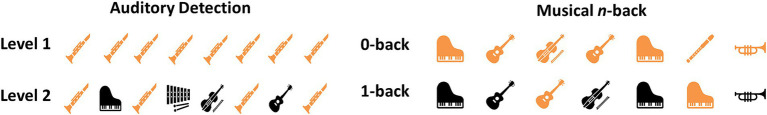
A visual representation of the two cognitive tasks used in this study is provided. Auditory Detection (left): Level 1 features the same melody played by the same musical instrument several times, and the participant is asked to click each time the melody is played. Level 2 presents melodies played by different instruments, and the participant is asked to click only when a melody by a specific instrument is played (in this example, the flute melody). Musical n-back (right): Levels 1 and 2 showcase melodies played by various instruments. In Level 1, the participant is asked to click whenever a melody is played, while in Level 2, the participant is asked to click only when a melody immediately repeats itself (regardless of which melody is played).

This study included a previously described auditory detection task (28), an auditory n-back task, and resting state tasks (see Figure 2).

The detection task included a sequence of tunes from a violin, trumpet, and flute. Participants held a clicker in order to respond to the musical cues. Instructions directed participants to click once when they heard a specific instrument playing. Responses were limited to “yes” trials corresponding to the designated instrument’s tune. The task was designed with two levels of difficulty to evaluate escalating cognitive demands. In Detection level 1, a consistent tune played for 3 s, recurring throughout the block. Participants were directed to click promptly for every repetition of the tune. This level featured three trials of 90 s each (corresponding to each instrument), with each melody appearing 5–6 times and intervals of 10–18 s of silence. Detection level 2 included tunes lasting for 1.5 s, of three instruments intertwined within a single block. Participants were instructed to respond solely to a designated instrument in that block, disregarding the rest. Each trial in this level had 6–8 melodies interspersed with 8–14 s of silence, and the target tune played 2–3 times.

In the n-back task, participants were presented with a sequence of melodies played by different instruments and used the clicker to respond to the stimuli. This task also included two difficulty levels (0-back and 1-back) to examine increasing cognitive load. A set of melodies (played by a violin, a trumpet, and a flute) was played in a different order for each block, and participants were asked to click a button when the melody repeated n-trials ago (based on the block level). In the 0-back level, participants clicked the button each time a melody was heard. This level included one 90-s block with 9 trials (instances of melody playing), each melody played for 1.5 s and 6–11 s of silence in between. In the 1-back level, participants clicked the button each time a melody repeated itself (*n* = 1). This level included two 90-s blocks with 12–14 trials (instances of melody playing), each melody played for 1.5 s and 4–6 s of silence in between. In each block, 30–40% of the trials were the target stimulus, where the melody repeated itself, and the participant was expected to click the button. The resting state tasks consisted of two blocks: one with 45 s and the other with 60 s of resting state recording.

Given the participants’ age and capabilities, a constraint for the experimental design was the session’s duration. Ensuring participant comfort and cooperation was paramount; thus, we maintained a tight protocol to include setup time, explanation, informed consent procedures, and the experiment within a 30-min window. This decision inevitably limited the number of trials per condition. However, as a countermeasure, we prioritized diversifying our conditions to facilitate robust cognitive load manipulations instead of simply increasing repetitions of the same condition.

#### 2.3.2 Signal processing

The EEG signal was decomposed into multiple components using harmonic analysis mathematical models (9, 33), and ML methods were employed on the components to extract higher-level EEG features. The full technical specifications for signal processing can be found in Molcho et al. (28). In summary, the Neurosteer® signal-processing algorithm analyzes EEG data using a time/frequency wavelet-packet analysis. This analysis, previously conducted on a separate dataset of EEG recordings, identified an optimal orthogonal basis decomposition from a large collection of wavelet packet atoms, optimized for that set of recordings using the Best Basis algorithm (34). This basis results generated a new representation of 121 optimized components called Brain Activity Features (BAFs). Each BAF consists of time-varying fundamental frequencies and their harmonics.

The BAFs are calculated over a 4-s window, which contains 2,048 time elements due to the 500 Hz sampling frequency. In this window, each BAF is a convolution of a time/frequency wavelet packet atom, allowing for a signal that can vary in frequency over the 4-s window, such as a chirp. The window is then advanced by 1 s, similar to a moving window spectrogram with 75% overlap, and the calculation is repeated for the new 4-s window. The EEG power spectrum is obtained using a fast Fourier transform (FFT) of the EEG signals within a 4-s window.

## 3 Results

### 3.1 Demographic results

To ensure that the groups were well-balanced, we compared some demographic characteristics of each group. First, for the positive vs. negative patients within the F-DOPA group, we compared the F-DOPA test age, symptoms onset age, difference between the F-DOPA test and the auditory assessment task (in years), and PD diagnosis age (in years), see Table 1 for details. No significant differences were found between these sub-groups (all *p*s > 0.05). Due to the small sample size of the negative F-DOPA group (*n* = 6), this analysis did not include a division between males and females. The mean time that had passed between the F-DOPA test (after which the diagnosis was determined) and the auditory assessment was 1.28 (1.37) years. Out of the F-DOPA group, 87% of the participants took PD medications during study completion. The most common medications were Azilect (31%), Amantadine (31%), Dopicar (25%), and Sinemet CR (15%); for the complete list of medications, see Supplementary material B and Table 1. For descriptive information about the motor symptoms, see Supplementary material B and Table 1.

**Table 1:** F-DOPA clinical information for the groups included in the first part of the analysis.

Study group	Gender	*n*	Auditory task age, years	F-DOPA test age, years	Symptoms onset age, years	F-DOPA – auditory task difference, years	PD diagnosis age, years
F-DOPA Negative	Female	4	61.77 (7.97)	62.03 (8.30)	57.79 (9.94)	0.49 (0.50)	62.02 (8.3)
	Male	2	74.50 (0.50)	75.01 (1.00)	74.50 (0.5)	0.5 (0.50)	75 (1)
F-DOPA Positive	Female	11	65.10 (13.10)	63.64 (12.85)	56.41 (12.72)	1.91 (1.73)	63.37 (12.73)
	Male	15	63.54 (11.22)	62.73 (11.41)	58.79 (11.59)	1.14 (1.10)	61.73 (12.03)

Averages are shown for males and females separately. No significant changes were found between the sub-groups for the measured parameters.

All recruited patients completed the auditory assessment tasks, and their EEG data was used. The average age of the PD patients group (*n* = 26, 11 Females) was 64.15 (12.30) years, and the average MMSE score was 29.61 (0.57). The average age of the Healthy group (*n* = 26, 11 Females) was 66.19 (6.49) years, and the average MMSE score was 29.07 (0.89). Overall, the mean age was 65.17 (9.79) years, with 42% females and 58% males. No significant differences in age or gender were found between the groups (all *p*s > 0.05). See Table 2 for complete demographic details and results.

**Table 2:** Demographic information for the groups included in the second part of the analysis.

	Groups	PD patients	Healthy
Total	*n*	26	26
	MMSE	29.61 (0.57)	29.07 (0.89)
	Age	64.15 (12.30)	66.19 (6.49)
	Age *t*-tests	PD patients vs. healthy: *t* = 0.54, *p* = 0.58	
Male	*n*	15	15
	MMSE	29.66 (0.61)	29.06 (0.88)
	Age	63.46 (11.61)	67.13 (5.89)
	Age *t*-tests	PD patients vs. healthy (male): *t* = 1.09, *p* = 0.28	
Female	*n*	11	11
	MMSE	29.54 (0.52)	29.09 (0.94)
	Age	65.09 (13.71)	64.90 (7.34)
	Age *t*-tests	PD patients vs. healthy (female): *t* = −0.03, *p* = 0.96	
Age males vs. females		*t* = −0.32, *p* = 0.74	*t* = 0.85, *p* = 0.39

Averages are shown for the total number of participants, as well as for males and females separately. *t* and *p* values of the comparisons between mean ages of the study groups are displayed for the total, and for males and females separately. Additionally, *t* and *p* values of the comparisons between age and MMSE scores for each gender are provided in the last rows.

### 3.2 Prediction model of F-DOPA results

For a full description of the prediction model methodology see Supplementary materials A. In the initial phase of data analysis, our primary objective was to develop a predictor capable of accurately classifying and predicting F-DOPA test results. The prediction model was formulated using machine learning (ML) methods applied to the extracted BAFs. As an integral component of the study design, one-third of the F-DOPA results were intentionally undisclosed to evaluate the prediction model. The process of developing such a predictor entails three steps: (1) identifying the feature representation from which the prediction is derived, (2) determining the type of data to be utilized in training the predictor, and (3) ascertaining the model family from which a predictor will be selected. In this pilot study, our primary focus was on identifying the type of representation that could yield a meaningful prediction. Consequently, we maintained the other two factors as constants, as detailed below.

To that end, we initially tested whether a feature representation based on connectivity between the BAFs was useful. Connectivity and causality Analysis had been used successfully in the context of neuroscience (35). In this study, we adopted the approach used by Friston et al. (35), applying it to the components extracted from single-channel EEG data rather than multiple electrodes or multiple fMRI regions (see full details in the Supplementary materials A). We employed connectivity-based representation and performed dimensionality reduction using principal components analysis (PCA) (36).

The PCA-derived reduced-dimensionality representation was used for training and testing the prediction model. We used a previously collected dataset, which included data from healthy participants and patients with PD performing similar auditory tasks, in conjunction with the positive F-DOPA labels collected in this study (*n* = 14) to serve as training data for the predictor. The testing data comprised of participants with undisclosed F-DOPA results (*n* = 12), participants with negative F-DOPA (*n* = 6), and healthy controls (*n* = 20).

The prediction model was based on an ensemble of ridge regression (37). Ridge regression extends linear regression by modifying the loss function to minimize the model’s complexity, introducing a constraint on the coefficients through a penalty factor equivalent to the square of the magnitude of the coefficients. The ensemble predictor consisted of 10 logistic regression predictors with regularization terms ranging from 1 to 10 (38). Studies have shown that ensembles with strong regularization values can mitigate noise in the data and produce better predictors (39).

The trained PCA model with ridge regression yielded a score between−1 and 1 for each participant, corresponding to a predicted test result label. A separating cutoff score of 0 was set, with data points higher than 0 classified as positive F-DOPA and those lower than 0 classified as negative F-DOPA. The prediction model labels were compared to the actual test labels to determine the model’s accuracy in classifying the 12 unknown patients and accurately classifying other groups as either negative or positive.

Due to a tendency for positive bias among patients referred for F-DOPA scans as part of standard care, the majority of collected F-DOPA results were positive. To include negative results, the six patients with negative F-DOPA results were also considered as part of the testing data. Since all 12 patients in the unknown group were eventually classified as positive, we performed additional quantitative analysis using Bayesian Mann–Whitney U Tests to determine the similarity between labeled groups. This follow-up analysis was conducted using a data augmentation algorithm with 5 chains of 1,000 iterations. We report the BF_01_ (i.e., the null hypothesis that H0 is not different from H1) of the Bayesian U tests between controls vs. negative and positive vs. unknown, and the BF_10_ (i.e., the hypothesis that H0 is different from H1) of the Bayesian U tests between control vs. positive and control vs. unknown groups. This analysis was performed using JASP 0.11.1.0 software (40) (JASP, Version 0.17).

#### 3.2.1 Prediction model results

**Figure 3:**
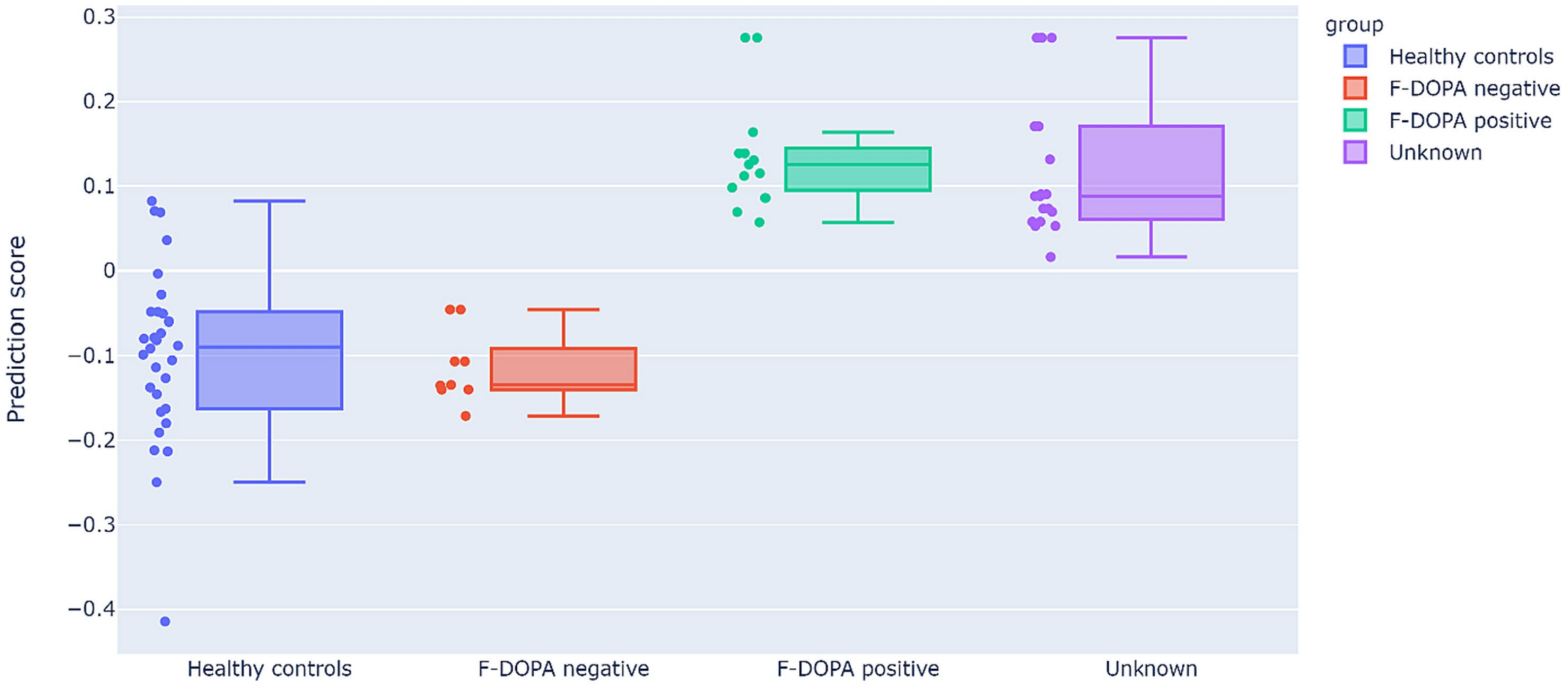
Results of the prediction model. The prediction scores (y-axis) cutoff between positive and negative labels is 0 (data points with prediction scores higher than 0 are classified as positive F-DOPA, whereas prediction scores lower than 0 are classified as negative F-DOPA). The study data are displayed in the graph as individual sample points and as F-DOPA groups: positive F-DOPA results, negative F-DOPA results, healthy age-matched controls, and initially unrevealed unknown F-DOPA results.

Figure 3 depicts the prediction model results. All 12 patients in the ‘Unknown’ group were classified as having a positive F-DOPA result based on the prediction model (i.e., all predictor results were > 0). Moreover, the predictor assigned negative values to the six patients initially labeled as negative F-DOPA and positive values to all 14 patients initially labeled as positive F-DOPA. The majority of the control group samples received negative values as expected, except for 4 samples in the control group (20%), who received a positive F-DOPA label. It would be of interest to follow these four individuals and test if there was a pre-symptomatic detection of dopamine depletion in these subjects.

Bayesian Mann–Whitney U tests revealed strong evidence that the predicted results of the control group differ from positive F-DOPA patients (BF_10_ = 121.88, W = 385, R^2^ = 1.04), and presented moderate evidence of similarity to the negatively labeled F-DOPA group (BF_01_ = 2.97, W = 116, R^2^ = 1.21). The group with unknown labels, who were all given positive predictor results, was strongly evident to differ from the control group (BF_10_ = 149.48, W = 550, R^2^ = 1.032), and showed moderate evidence of similarity to the positive group (BF_01_ = 2.145, W = 98, R^2^ = 1). For all U-test outputs and figures, see Supplementary materials B.

### 3.3 High-level features and mixed linear models (LMM) analysis

The second part of data analysis was focused on evaluating the ability of previously extracted high-level EEG features and conventional frequency bands to differentiate between PD patients and healthy controls based on the auditory assessment protocol. A significant advantage of utilizing Linear Mixed Models (LMM) in our analysis stems from its capability to leverage multiple data points from each subject. Specifically, in our experiment, each second of activity represented a data point. By incorporating these into the model as random factors, LMM can effectively counteract the limitations posed by the reduced number of trials per condition (i.e., cognitive load level). This methodological approach optimally leverages our data structure, allowing for meaningful statistical interpretations despite the constraints on session duration and number of repetitions.

#### 3.3.1 High-level EEG features

The EEG features were previously generated using machine learning (ML) techniques applied to the Brain Activity Features (BAFs) from labeled datasets previously collected by Neurosteer. Specifically, EEG features A0 and L1, employed in this study, were calculated using the linear discriminant analysis (LDA) technique (41). The LDA technique aims to identify an optimal linear transformation that maximizes class separability.

Data analysis included the activity of EEG features A0 and L1, normalized to a scale of 0–100. The EEG variables were calculated every second from a moving window of 4 s, and the mean activity per condition was incorporated into the analyses.

#### 3.3.2 Frequency bands

The EEG dependent variables incorporated the power spectral density. Absolute power values were converted to logarithm base 10, resulting in values expressed in dBμV. Among the frequency bands, Delta (0.5–4 Hz) and Theta (4–7 Hz) were included. Preliminary tests indicated that the other frequency bands, such as Alpha (8–15 Hz), Beta (16–31 Hz), and lower Gamma (32–45 Hz), did not demonstrate any significant correlations or differences in the current data.

#### 3.3.3 LMM analysis comparing PD patients and healthy controls

In order to detect differences between PD patients and healthy controls, we employed a general linear mixed model (GLMM) (42), which incorporates both fixed and random effects. This model was preferred over the simpler GLM due to the relatively small sample size, as the GLMM accounts for the random slope for each participant. The model included the fixed within-participant variable of cognitive load level, as well as the group as a between-participants variable.

The group variable consisted of two levels: ‘PD Patients’ (patients with positive F-DOPA results) and ‘Healthy Controls’ (comprising both patients with negative F-DOPA results and healthy age-matched controls). As an initial validation, student t-tests were performed on each EEG variable between the subjects in the ‘Healthy Controls’ group to ensure there were no inherent differences in EEG activity between the two sub-groups (i.e., patients with negative F-DOPA results vs. healthy age-matched controls).

The cognitive load variable was an ordinal variable, coded linearly according to the task cognitive load level (from low to high) as follows: resting state = 0; detection level 1 = 1; 0-back = 1; detection level 2 = 2; and 1-back = 2. The model included the samples per participant per task (i.e., samples per second of activation) as a random slope. For models that demonstrated a significant main effect of cognitive load, post-hoc analyses were conducted, comparing possible pairwise combinations of cognitive load levels for each group (i.e., healthy vs. PD), using the Benjamini-Hochberg correction (43) for multiple comparisons. The significance level for all analyses was set to *p* < 0.05. All analyses were conducted using RStudio version 1.4.1717 (44).

#### 3.3.4 LMM results – comparing EEG variables between PD patient and healthy controls

##### 3.3.4.1 Initial validation

To rule out any intrinsic differences within the ‘healthy controls’ group, we compared between the subgroups composing the group: patients with negative F-DOPA results and the healthy age-matched patients. No significant differences were found for any of the EEG variables (*p* = 0.249, *p* = 0.64, *p* = 0.3 and *p* = 0.406 for Delta, Theta, A0 and L1, respectively).

##### 3.3.4.2 LMM models

**Figure 4:**
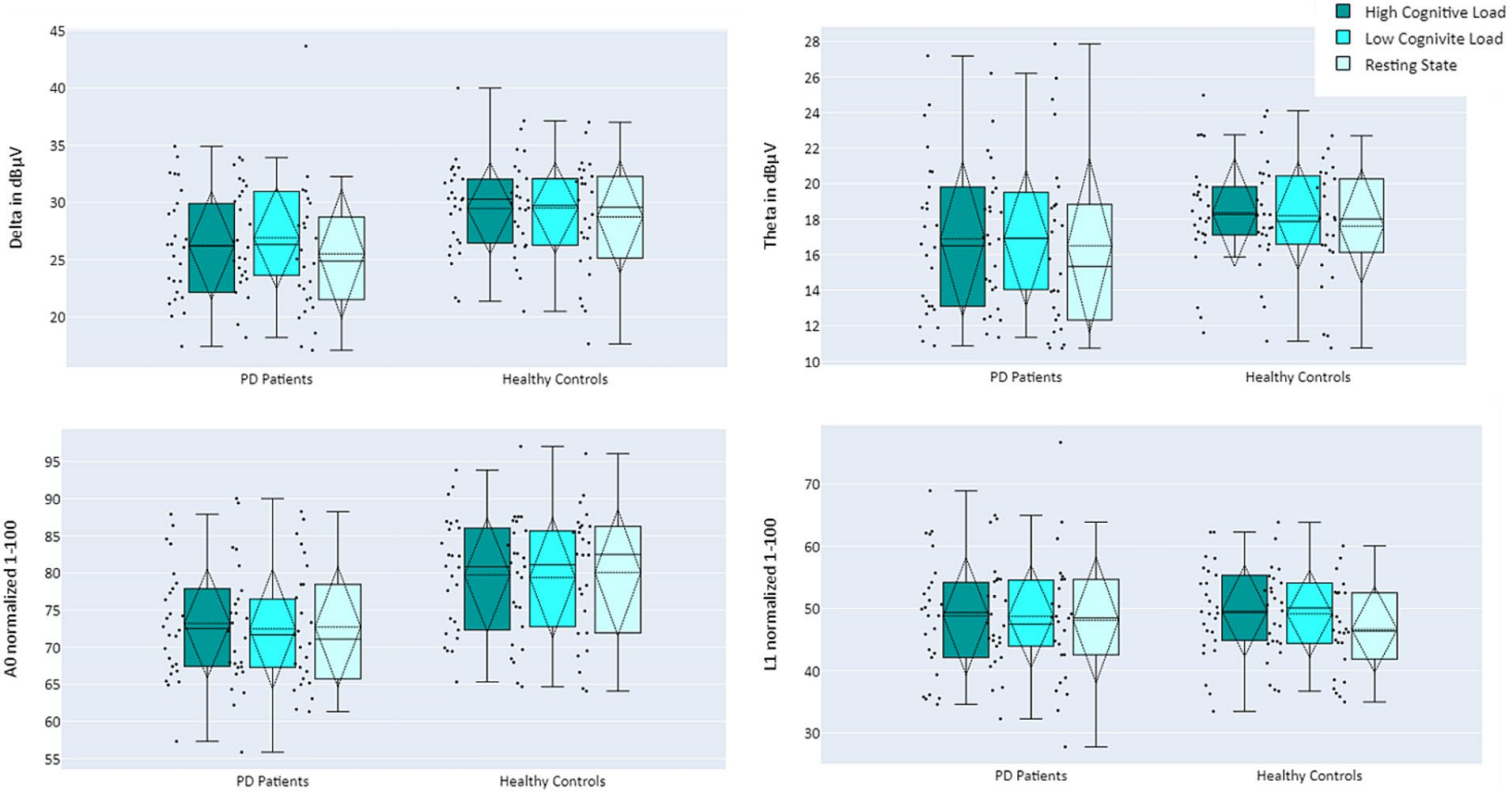
Mean activity of EEG features Delta (top left), Theta (top right), A0 (bottom left), and L1 (bottom right), comparing PD Patients (left) and Healthy Controls (right) during performance of cognitive tasks, as a function of cognitive load: high cognitive load (dark turquoise), low cognitive load (turquoise), and resting state (light turquoise).

The activity of EEG features per participant, as a function of group and cognitive load, is presented in Figure 4. For a full description of the models’ outputs see Table 3. Delta and A0 showed higher mean activity for the healthy controls compared to the PD patients (*p* = 0.01 and *p* = 0.003, respectively). Cognitive load ordinal effect reached significance for L1 (*p* = 0.043). Paired t-test analysis revealed that for the healthy group, L1 activity during the resting state task was significantly lower than during the high-cognitive load condition (adjusted *p* = 0.022), whereas in the PD group, no significant difference was found in L1 activity between any of the cognitive load conditions (see Table 4; Figure 4).

**Table 3:** Fixed effect coefficients, standard error, *z*-values, *p*-values, and 95% confidence interval outputs from the LMMs conducted on EEG features, with group (healthy controls vs. Parkinson’s patients), and cognitive load (resting state vs. low-load vs. high-load) coded as numeric variable.

	Fixed effect	Coef.	Std.Err.	z	*p* > |z|	[0.025	0.975]
Delta	Intercept	2.24	0.87	2.58	0.01	0.54	3.94
	Group	2.99	1.16	2.58	**0.01**	0.72	5.25
	Cognitive load	0.12	0.26	0.45	0.651	−0.39	0.62
Theta	Intercept	−7.38	0.69	−10.70	<0.001	−8.73	−6.03
	Group	1.24	0.96	1.28	0.199	−0.65	3.12
	Cognitive load	0.21	0.17	1.21	0.226	−0.13	0.54
A0	Intercept	72.72	1.59	45.74	<0.001	69.60	75.84
	Group	6.30	2.13	2.97	**0.003**	2.14	10.47
	Cognitive load	0.24	0.21	1.13	0.258	−0.18	0.66
L1	Intercept	48.17	1.51	31.84	<0.001	45.20	51.13
	Group	−0.30	2.15	−0.14	0.889	−4.51	3.91
	Cognitive load	0.65	0.32	2.02	**0.043**	0.02	1.28

Bold values represent significant effects (*p* < 0.05).

**Table 4:** *t* values, *p* values and *p* BH adjusted values of the pairwise comparisons of the L1 activity per each group, between the three cognitive load conditions: high-load, low-load, and resting state.

Group	Comparison	*t* value	*p* value	*p* adj BH
Healthy controls L1 activity	(high, low)	2.92	0.007	**0.022**
	(mid, low)	2.11	0.045	0.068
	(mid, high)	−0.61	0.544	0.544
PD Patients L1 activity	(high, low)	0.60	0.549	1.649
	(mid, low)	0.52	0.607	0.911
	(mid, high)	−0.08	0.933	0.933

Bold values represent significant effects (*p* < 0.05).

## 4 Discussion

While the F-DOPA PET scan is acknowledged for its diagnostic utility in parkinsonism (45), it is constrained by its fiscal demands and invasiveness, making the identification of valid biomarkers for early diagnosis and disease progression in PD vital (46). This pilot study utilized a single-channel EEG device, combined with signal processing, connectivity analysis, and machine learning-derived features, to recognize activity pattern differences between positive and negative F-DOPA test subjects. The ML predictor presented here effectively identified all unrevealed F-DOPA scores and 80% of the control cohort. Moreover, at the group level, previously extracted biomarkers applied to this dataset, highlighted differences in group averages (features Delta and A0) and irregular resting state neural activity in positive F-DOPA patients (feature L1).

The predictor accurately determined that 12 of the unrevealed test results were properly categorized as positive F-DOPA scores. In addition, it correctly detected the outcomes of both the negative and the positive F-DOPA scores that were initially disclosed. Moreover, most of the control group received a negative label as expected, except for 4 samples in the control group (20%), who received positive F-DOPA labels. This may be within the margin of error, or these participants may be prodromal PD patients. A follow-up longitudinal study following these patients would be beneficial to corroborate this. Nevertheless, the preliminary prediction results presented here support the notion of the power of prediction for individual patients rather than analysis of a group of patients. The supplementary Bayesian analysis further substantiated the predictor’s accuracy, providing evidence that the predictor output for F-DOPA-positive patients significantly diverged from that of healthy controls and the patients with negative results. In contrast, the output for F-DOPA-positive patients was indistinguishable from that of the 12 previously unknown patients, all subsequently confirmed as F-DOPA-positive. As the predictor was specifically engineered to distinguish between F-DOPA-positive and F-DOPA-negative outcomes, no notable differences were observed between the F-DOPA-negative patients and the control group participants. Nonetheless, the possibility of existing disparities in brain activity between the F-DOPA-negative patients who were referred to perform an F-DOPA test due to some clinical manifestations and the healthy controls cannot be dismissed, and the brain activity of the symptomatic F-DOPA negative group should be further investigated. This notion is supported by the raw data underlying the predictor’s development, as depicted in Supplementary materials A.

Upon revealing all F-DOPA results, further analysis was conducted to compare the EEG activity of PD patients vs. healthy controls, as well as cognitive load levels (manipulated by different auditory cognitive tasks). Results indicate that the Delta band and EEG feature A0 differentiate between the groups: Delta and A0 exhibited lower activity for the PD patients compared to healthy controls. This difference was more pronounced for A0 than Delta, suggesting that A0 may be more sensitive to functional changes, a notion supported by the highest separation demonstrated between groups with different levels of cognitive decline in a previous study (28). Finally, the L1 biomarker, which was previously shown to correlate with cognitive load (29), exhibited lower activity in resting state for healthy controls. In contrast, PD patients did not display such a decrease in L1 activity in the resting state condition. This finding aligns with previous research regarding resting state activity within PD patients. PD is characterized by higher resting EEG total power compared to healthy controls and slower oscillations in brain activity during resting state – a phenomenon independent of the disease’s stage, duration, and severity, and is also resistant to treatment with dopamine (47, 48). In conjunction, activation patterns of the two biomarkers – a decrease in A0 mean activity and no difference in L1 between high cognitive load and rest may serve as early indications of PD.

Despite the promising initial results, this study has several limitations. The generalization of the results is restricted due to the small sample size, and further studies with larger cohorts of patients are necessary to validate these preliminary findings. Investigations comparing various other indications for PD, including cerebrospinal fluid (CSF) and blood biomarkers, would also be beneficial in validating the EEG biomarkers and their predictive power. Moreover, the absence of detailed clinical information precludes the performance of in-depth EEG-clinical correlations; We do not have enough data to design a clinical profile of the negative F-DOPA group. Follow-up studies testing the symptomatic patients who received a negative F-DOPA result, as well as the four healthy patients, would greatly contribute to a better understanding of the results. Additionally, we did not perform an analysis in accordance with medication treatment and clinical symptoms of our patients. Our approach employs wavelet-packet analysis as a pre-processing step for ML, creating components composed of time-varying fundamental frequencies and their harmonics. These complex time/frequency components of dynamic nature are instrumental in the interpretation of the EEG signal. Future research should explore the utility of this approach in the assessment of neurological disorders. Additionally, examining the potential usefulness of the EEG features presented here in controlled studies characterizing EEG signal changes in seniors may contribute to understanding the association of these features with basic brain function. This warrants further investigation to evaluate the single-channel EEG with ML analysis as a potential new biomarker in the context of PD.

The fact that a single-channel EEG with auditory cognitive assessment was able to differentiate between patients with positive vs. negative F-DOPA PET results may support the hypothesis that a single-channel EEG could reflect the dopaminergic function of the brain. Furthermore, while F-DOPA PET is based on metabolic function and predominantly reflects dopaminergic deficit, EEG data may potentially represent functional disability due to dopaminergic deficit. Discrimination based on features extracted from a single EEG channel could potentially lead to an objective physiological assessment to aid in the early detection and diagnosis of PD.

## Data availability statement

The datasets presented in this article are not readily available because of ethical and privacy restrictions. Requests to access the datasets should be directed to NI, nathan@neuroteer.com.

## Ethics statement

The studies involving humans were approved by Tel Aviv Sourasky Medical Center; Dorot Geriatric Medical Center. The studies were conducted in accordance with the local legislation and institutional requirements. The participants provided their written informed consent to participate in this study.

## Author contributions

LM: Conceptualization, Data curation, Formal analysis, Investigation, Methodology, Project administration, Writing – original draft, Writing – review & editing. NM: Conceptualization, Formal analysis, Investigation, Methodology, Writing – original draft, Writing – review & editing. NH: Data curation, Investigation, Writing – original draft. TZ: Data curation, Formal analysis, Investigation, Writing – original draft, Writing – review & editing. NI: Conceptualization, Formal analysis, Investigation, Methodology, Project administration, Software, Supervision, Writing – original draft, Writing – review & editing. TG: Conceptualization, Investigation, Methodology, Supervision, Writing – original draft, Writing – review & editing.
