# Supplementary material for: Evaluation of Parkinson’s disease early diagnosis using single-channel EEG features and auditory cognitive assessment
Source: Front Neurol. 2023 Dec 19;14:1273458. doi: 10.3389/fneur.2023.1273458 (PMC10762798; doi:10.3389/fneur.2023.1273458)
Supplement: Supplementary file 1 [file Data_Sheet_1.pdf]

frontiers

Supplementary Material A

Evaluation of Parkinson's disease early diagnosis using single-channel EEG features and auditory cognitive assessment

Lior Molcho[†1*], Neta B. Maimon[†1,2], Neomi Hezi[4], Talya Zeimer[1], Nathan Intrator[1,3,4], Tanya Gurevich[4,5,6]

[1] Neurosteer Inc, NYC, New York, USA

[2] School of Psychological Sciences, Tel Aviv University, Tel Aviv, Israel

[3] Blavatnik School of Computer Science, Tel-Aviv University, Tel-Aviv, Israel;

[4] Sagol School of Neuroscience, Tel Aviv University, Tel Aviv, Israel

[5] Movement Disorders Unit, Neurological Institute , Tel Aviv Sourasky Medical Center.

[6] Faculty of Medicine, Tel-Aviv University, Israel

Prediction model

Background

In the context of neuroscience, connectivity analysis was proposed to study the correlation or causality of BOLD activity between different brain regions (1). It was later extended to measure the correlation and causality of EEG brain activity between different electrodes (2), and further extended to perform the connectivity between electrodes after undergoing an independent components analysis (3), source localization analysis (4), or other multiple electrodes transformation such as spectral PCA (5). Thus, connectivity analysis has been done so far between different fMRI regions, or different EEG electrodes, and in general, between multiple sources. Here we present connectivity analysis between components obtained from a single source - the same EEG channel.

In Parkinson's disease (PD), connectivity analysis proved to be a useful tool to establish underlying pathophysiology and network connectivity related to the motor and non-motor symptoms of the disease. Multiple studies using fMRI-based connectivity analysis give insight into disrupted connectivity in the PD patient population. An abnormal activation of different areas of the motor and resting state networks in patients with early and late-stage PD was shown to be related to cardinal clinical features. For example, it was shown that Parkinson's tremor-related activity first arises in the basal ganglia and is then propagated to the cerebello-thalamo-cortical circuit, determining the role of those circuits in initiating and maintaining tremor symptoms (6). Another study suggests that some of the factors related to PD patients having difficulty achieving automatic movement are less efficient neural coding of movement and failure to shift execution of automatic movements more subcortically, and these changes of effective connectivity become more abnormal as the disorder progresses (7). Additionally, a great body of evidence exists in the literature discussing the decreased functionality of the default mode network (DMN) as part of PD progression. For example, resting

state fMRI connectivity revealed a disrupted functional integration in cortico-striatal loops in the sensorimotor network in patients with PD (8). Decreased functional connectivity in mesolimbic-striatal and cortico-striatal loops was found in drug-naïve PD patients compared to healthy controls (9). It has also been shown that PD patients with cognitive impairment predominantly showed a reduced connectivity in specific brain regions that are part of the default mode network (10).

EEG connectivity studies have also demonstrated functional connectivity disruptions in PD patients. A recent review exploring over 85 such studies concluded that the main observations were a general slowing of background activity, excessive synchronization of beta activity, and disturbed movement-related gamma oscillations in the basal ganglia and in the cortico-subcortical and cortico-cortical motor loops (11).

Methods

The prediction model used in this study presents connectivity analysis based on a single EEG source. This is done by first decomposing the signal into multiple components via a time-frequency optimal orthogonal decomposition, and then, performing connectivity analysis on these components. We performed an orthogonal decomposition using the Best Basis Algorithm (12) as described in Molcho et al., 2022 (13).

We follow the methodology of BOLD connectivity analysis as described in Fristen et al., 1996 (14), but use it on the components decomposed from the single-channel EEG. These components were extracted from earlier collected data of multiple subjects performing multiple tasks. The observations were in the form of 4-second time windows with a 3 second overlap. Projecting the 121 components previously extracted on the data collected in this study lead to 121 time series that are updated once per second. From these time series, we obtain a 121x121 connectivity matrix. The matrix can be symmetric and indicate correlations between the different time series or non-symmetric and indicate directed causality between the components. Here we focus on the symmetric correlation matrix.

The matrix of correlations that is obtained from each individual patient can be used as an input to a machine learning based predictor that is trained by labeled data previously collected (Figure 3 in manuscript). It can also be used for a group analysis and visualization of the connectivity of each group of patients (see Supplementary Figure 1). To obtain patient population connectivity, we averaged the correlation matrix over the group members with a specific clinical diagnosis. Given an averaged matrix of correlations between different components, a classical connectivity representation (15) is not possible due to the large number of connections to present: 121x60. We resolve this by converting the correlation matrix into a matrix of distances between components, such that higher correlation reflects a smaller distance, by using:

$$d_{ij} = e^{-\mathrm{corr}\,(c_i\,,c_j)}$$

Then, we project the high dimension space of all correlations (121x120/2 dimensions) onto a two-dimensional space while preserving the local distance between every two components as much as possible. This is done via multi-dimensional scaling (MDS) as suggested for fMRI by Friston et al., 1996 (14). The correlation between different regions is calculated and then projected via MDS to obtain a distance map that is implied by the correlations. The projection via MDS, provides a practical visualization for the connectivity of a large number of nodes, which in our case, refer to the 121 components extracted from the single-channel EEG.

Results

Supplementary Figure 1 presents the connectivity maps based on the MDS representation described above. The figure contains the 121 components included in the analysis (extracted from the single-channel EEG). They are marked by numbers on the graphs and corresponding heat-map colors. The distances between each component to another, represent the correlation between them as obtained by the model. A lower absolute distance between two components in the graph represents higher connectivity between those two components.

The figure presents 3 different groups of patients: positive F-DOPA (left, n=24), Negative F-DOPA (middle, n=6), and healthy age-matched controls (right, n=24). A clear component connectivity difference between the F-DOPA positive group and the healthy age-matched control group is visible. Interestingly, the F-DOPA negative patients' connectivity map appears closer to the healthy group in correlation between the components, but with distinct changes which should be further explored.

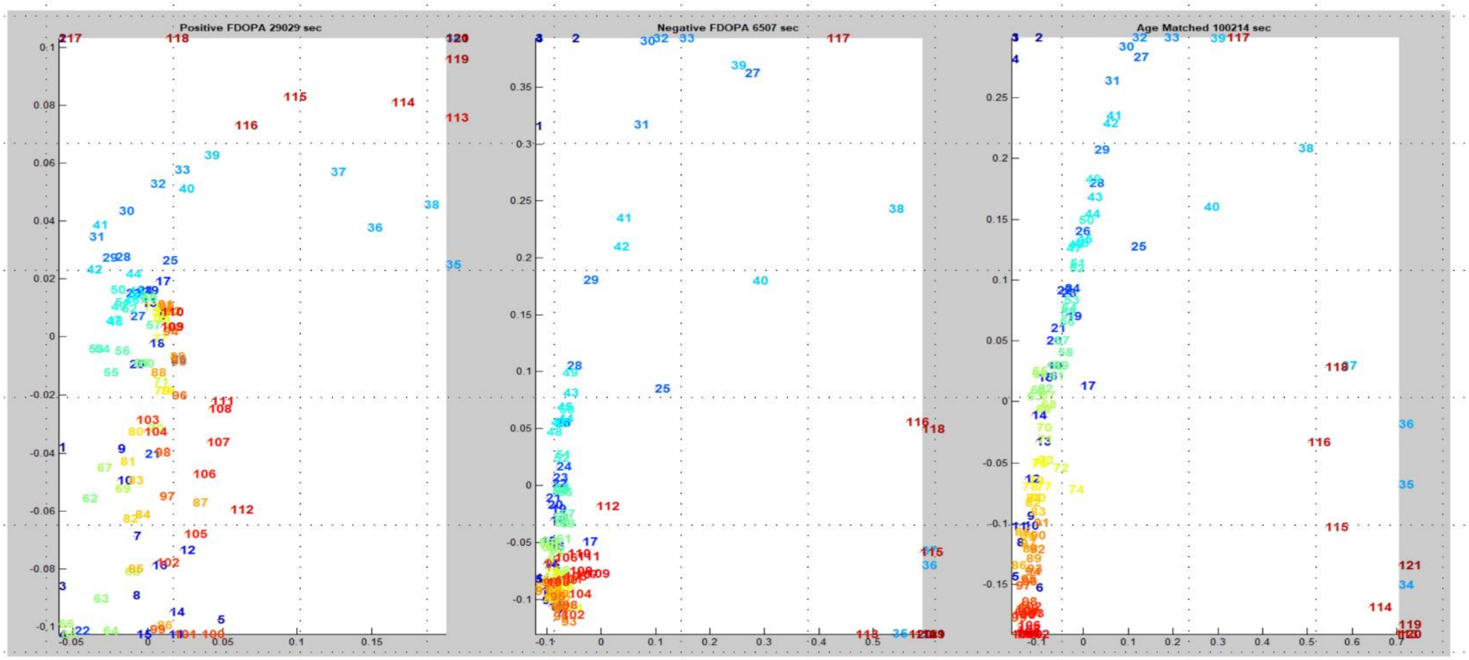

Supplementary Figure 1. Group connectivity representation between components extracted from a single-channel EEG, showing proximity of the components, in three groups: F-DOPA positive patients (left), F-DOPA negative patients (middle), and healthy age-matched subjects (right). The numbers represent the component number from the 121 component representation and for better visibility the numbers are also colored using the heat color map so that larger channel-numbers are red and lower channel-numbers are blue. Note that lower distance represents higher connectivity between two components. The axes of the representation have no real meaning and represent the two dimensions obtained from the optimization process (the MDS) to embed the total number of correlations onto a two-dimensional space.

F-DOPA classification Prediction

Supplementary Figure 1 demonstrated the difference in connectivity between three groups of subjects. However, while the group difference may be very evident, an important question is whether this difference can be translated into individual differences, which can lead to a classifier of individual subjects. To this end, we created a classifier using age-matched individuals that were not diagnosed with Parkinson's disease, and 14 F-DOPA-positive patients. We then tested the classifier on 18 subjects, of which 6 were F-DOPA negative and 12 F-DOPA positive. The results are depicted in Figure 3 of the manuscript.

The input to the predictor is the collection of correlations between the different EEG components for each individual. These correlations are calculated during the 12-minute cognitive assessment that the patients performed. This collection of correlations is of dimension 121x120/2 which is 7260. Thus, to avoid overfitting and obtain a smaller predictor, we performed an unsupervised dimensionality reduction based on previously collected data. Specifically, using Principal Components Analysis (PCA) (16), we created a standard dimensionality reduction that is used in different studies, from data of young and senior subjects all performing the same cognitive task (13,17). This corresponds to step A in the construction of the classifier (See Supplementary Figure 2).
Step B trains a set of logistic regression classifiers. The training data as mentioned before included 14 F-DOPA positive and 24 age-matched F-DOPA negative patients. The regressors have different ridge regularizes. We then ensemble average all the regressors (18) to obtain the final predictor.
Step C applies the obtained set of classifiers on the test data, which included 12 F-DOPA positive results and 6 F-DOPA negative results and 30 records from 20 healthy age-matched controls. First the data is projected on the previously calculated. dimensionality reduction and then passed by the full set of regressors.

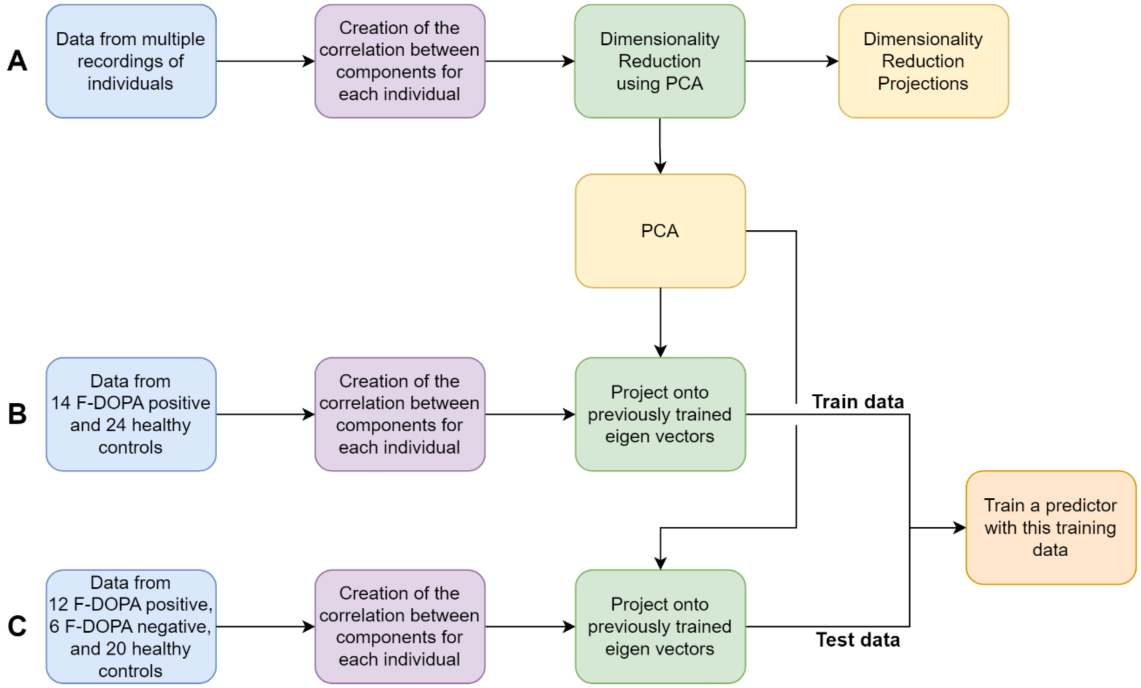

Supplementary Figure 2. Scheme of the classification prediction model calculations.

References

1. Rogers BP, Morgan VL, Newton AT, Gore JC. Assessing functional connectivity in the human brain by fMRI. Magn Reson Imaging. 2007;25(10).

2. Schoffelen JM, Gross J. Source connectivity analysis with MEG and EEG. Vol. 30, Human Brain Mapping. 2009.

3. Chen JL, Ros T, Gruzelier JH. Dynamic changes of ICA-derived EEG functional connectivity in the resting state. Hum Brain Mapp. 2013;34(4).

4. Dai Z, De Souza J, Lim J, Ho PM, Chen Y, Li J, et al. Eeg cortical connectivity analysis of working memory reveals topological reorganization in theta and alpha bands. Front Hum Neurosci. 2017;11.

5. Smith EE, Bel-Bahar TS, Kayser J. A systematic data-driven approach to analyze sensor-level EEG connectivity: Identifying robust phase-synchronized network components using surface Laplacian with spectral-spatial PCA. Psychophysiology. 2022;59(10).

6. Dirkx MF, den Ouden H, Aarts E, Timmer M, Bloem BR, Toni I, et al. The cerebral network of parkinson's tremor: An effective connectivity fMRI study. Journal of Neuroscience. 2016;36(19).

7. Wu T, Chan P, Hallett M. Effective connectivity of neural networks in automatic movements in Parkinson's disease. Neuroimage. 2010;49(3).

8. Tessitore A, Giordano A, de Micco R, Russo A, Tedeschi G. Sensorimotor connectivity in Parkinson's disease: The role of functional neuroimaging. Front Neurol. 2014;5(SEP).

9. Luo CY, Song W, Chen Q, Zheng ZZ, Chen K, Cao B, et al. Reduced functional connectivity in early-stage drug-naive Parkinson's disease: A resting-state fMRI study. Neurobiol Aging. 2014;35(2).

10. Wolters AF, van de Weijer SCF, Leentjens AFG, Duits AA, Jacobs HIL, Kuijf ML. Resting-state fMRI in Parkinson's disease patients with cognitive impairment: A meta-analysis. Vol. 62, Parkinsonism and Related Disorders. 2019.

11. Bočková M, Rektor I. Impairment of brain functions in Parkinson's disease reflected by alterations in neural connectivity in EEG studies: A viewpoint. Vol. 130, Clinical Neurophysiology. 2019.

12. Coifman RR, Meyer Y, Quake S, Wickerhauser MV. Signal processing and compression with wavelet packets. In: Wavelets and Their Applications. 1994.

13. Molcho L, Maimon NB, Regev-Plotnik N, Rabinowicz S, Intrator N, Sasson A. Single-Channel EEG Features Reveal an Association With Cognitive Decline in Seniors Performing Auditory Cognitive Assessment. 2022; Available from: www.frontiersin.org

14. Fristen KJ, Frith CD, Fletcher P, Liddle PF, Frackowiak RSJ. Functional topography: Multidimensional scaling and functional connectivity in the brain. Cerebral Cortex. 1996;6(2).

15. Lv H, Wang Z, Tong E, Williams LM, Zaharchuk G, Zeineh M, et al. Resting-state functional MRI: Everything that nonexperts have always wanted to know. Vol. 39, American Journal of Neuroradiology. 2018.

16. Clarke MRB, Duda RO, Hart PE. Pattern Classification and Scene Analysis. J R Stat Soc Ser A. 1974;137(3).

17. Bolton F, Te'Eni D, Maimon NB, Toch E. Detecting interruption events using EEG. In: 2021 IEEE 3rd Global Conference on Life Sciences and Technologies (LifeTech). IEEE; 2021. p. 33–4.

18. Raviv Y, Intrator N. Bootstrapping with Noise: An Effective Regularization Technique. Conn Sci. 1996;8(3–4).
