# Supplementary material for: Evaluation of Parkinson’s disease early diagnosis using single-channel EEG features and auditory cognitive assessment
Source: Front Neurol. 2023 Dec 19;14:1273458. doi: 10.3389/fneur.2023.1273458 (PMC10762798; doi:10.3389/fneur.2023.1273458)
Supplement: Supplementary file 2 [file Data_Sheet_2.pdf]

Supplementary Material B

Evaluation of Parkinson's disease early diagnosis using single-channel EEG features and auditory cognitive assessment

Lior Molcho[†1*], Neta B. Maimon[†1,2], Neomi Hezi[5], Talya Zeimer[1], Nathan Intrator[1,3,4], Tanya Gurevich[4,5,6]

[1] Neurosteer Inc, NYC, New York, USA

[2] School of Psychological Sciences, Tel Aviv University, Tel Aviv, Israel

[3] Blavatnik School of Computer Science, Tel-Aviv University, Tel-Aviv, Israel;

[4] Sagol School of Neuroscience, Tel Aviv University, Tel Aviv, Israel

[5] Movement Disorders Unit, Neurological Institute , Tel Aviv Sourasky Medical Center.

[6] Faculty of Medicine, Tel-Aviv University, Israel

Demographic results

To ensure that the groups were well-balanced we compared some demographic characteristics of each group. Table 1 presents descriptive information regarding the motor symptoms side. The table presents the group association, gender and laterality of the motor symptoms of the patients. Data of 29 patients was collected, 3 additional patients did not have this data in their record.

Study group	PD medications	Count
Negative	Sinemet CR (Levodopa / carbidopa)	1
	Propranolol	1
	Rasagiline	1
	Amantadine	1
Positive	Sinemet CR (Levodopa / carbidopa)	4
	Rasagiline	9
	Amantadine	8
	Levodopa/ carbidopa/entacapone	2
	levodopa/carbidopa	8
	levodopa/ benserazide	1

	Pramipexol	2
	Losartan + Hydrochlorothiazide	1
	ISOSORBIDE MONONITRATE	1
	Propranolol	1
	Gabapentin	1
	Mirtazapine	1
	Aspirin	1
	Rasagiline	1
	Simvastatin	1
	Losartan	1
	Trihexyphenidyl	1

Table 1. PD medications given at the time of the auditory test for patients holding a valid F-DOPA test result.

Study group	Gender	Motor symptom side	Count
Negative	Female	Bilateral	1
		L	1
		R	2
	Male	Bilateral	1
Positive	Female	Bilateral	4
		L	2
		R	5
	Male	Bilateral	4
		L	2
		R	7

Table 2. Motor symptom laterality information for patients holding a valid F-DOPA test result.

Bayesian results

Quantitative analysis using Bayesian Mann-Whitney U tests was performed to determine the similarity between the groups. This follow-up analysis was conducted using a data augmentation algorithm with 5 chains of 1000 iterations. We report the BF_{01} (i.e., the null hypothesis that H0 is not different from H1) of the Bayesian U tests between controls vs. negative; and positive vs. unknown, and the BF_{10} (i.e., the hypothesis that H0 is different from H1) of the Bayesian U tests between control vs. positive; and control vs. unknown groups.

Bayesian Mann-Whitney U tests revealed strong evidence that the predicted results of the control group differ from positive F-DOPA patients (BF_{10} = 121.88, W = 385, R^2 = 1.04), and presented moderate evidence of similarity to the negatively labeled F-DOPA group (BF_{01} = 2.97, W = 116, R^2 = 1.21). The group with unknown labels, who were all given positive predictor results, was strongly evident to differ from the control group (BF_{10} = 149.48, W = 550, R^2 = 1.032), and showed moderate evidence of similarity to the positive group (BF_{01} = 2.145, W = 98, R^2 = 1). The U tests outputs and figures are presented in tables 2-5 and figures 1-4.

	BF_{01}	W	R^2
Prediction healthy vs. negative	2.793	116.000	1.028

Table 2. Bayesian Mann-Whitney U test for prediction healthy vs. negative groups.

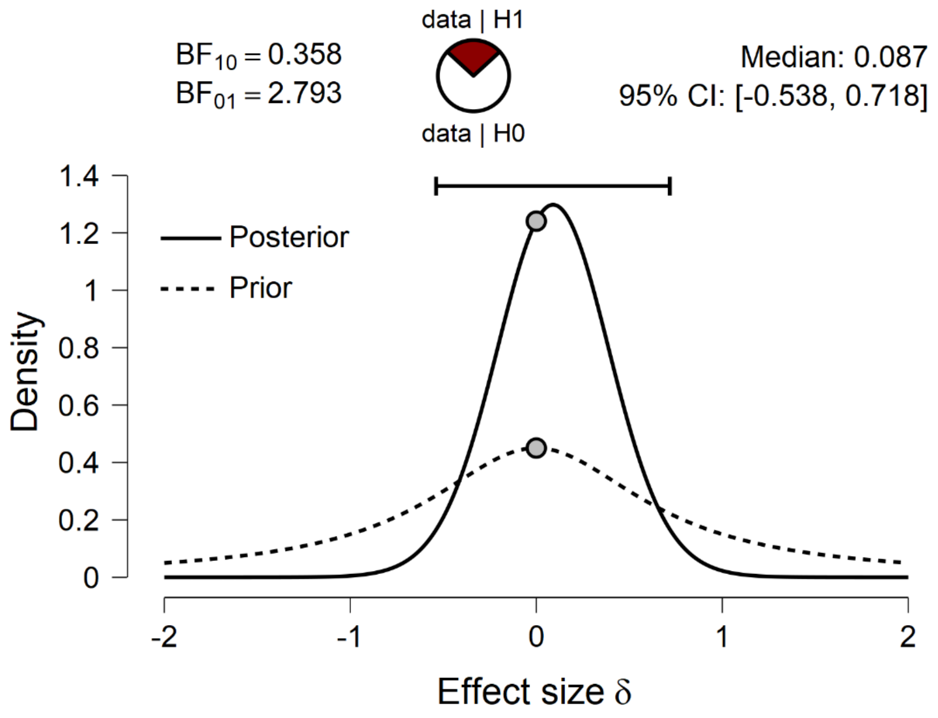

Figure 1. Inferential plots prediction healthy vs. negative groups prior and posterior.

	**BF_{10}**	**W**	**R^2**
Prediction healthy vs. positive	121.881	385.000	1.043

Table 3. Bayesian Mann-Whitney U test of prediction healthy vs. positive groups.

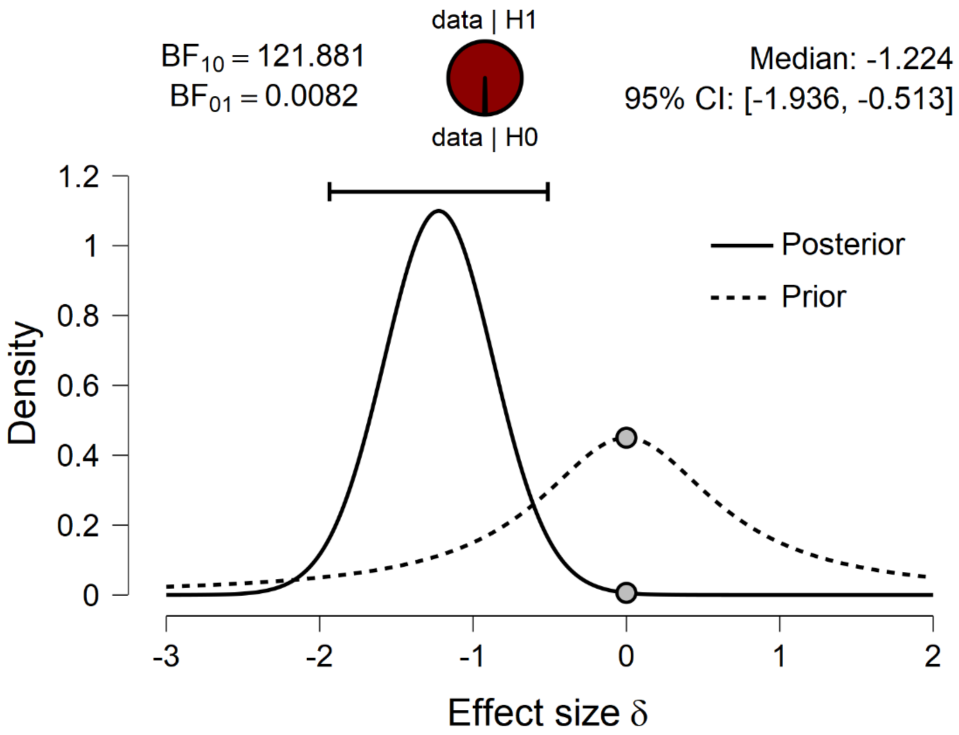

Figure 2. Inferential plots prediction healthy vs. positive groups prior and posterior.

	BF_{10}	W	R^2
Prediction healthy vs. unknown	149.481	550.000	1.032

Table 4. Bayesian Mann-Whitney U test for prediction healthy vs. unknown groups.

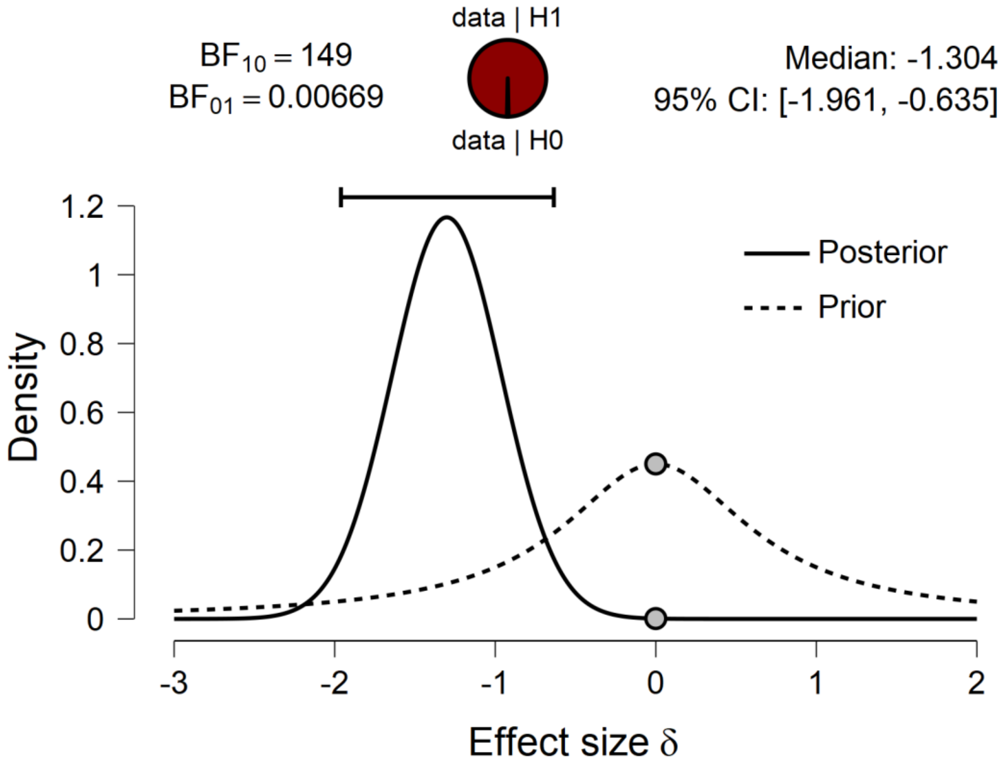

Figure 3. Inferential plots prediction healthy vs. unknown groups prior and posterior.

	BF_{01}	W	R^2
Prediction positive vs. unknown	2.145	98.000	1.000

Table 5. Bayesian Mann-Whitney U test for prediction positive vs. unknown groups.

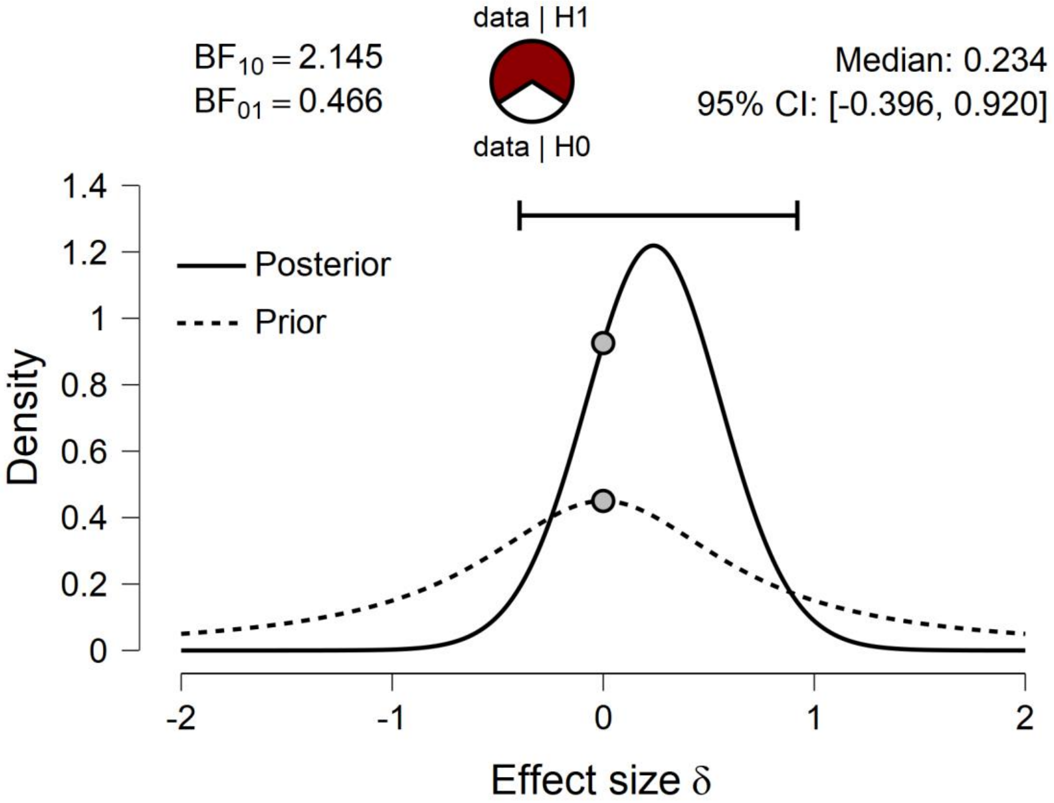

Figure 4. Inferential plots prediction positive vs. unknown groups prior and posterior.
