# Supplementary material for: Evaluation of Parkinson’s disease early diagnosis using single-channel EEG features and auditory cognitive assessment
Source: Front Neurol. 2023 Dec 19;14:1273458. doi: 10.3389/fneur.2023.1273458 (PMC10762798; doi:10.3389/fneur.2023.1273458)
Supplement: Supplementary file 3 [file Data_Sheet_3.pdf]

F-DOPA Group

In [50]:
```
df_F_DOPA_gen = df_F_DOPA[['f_dopa_group', 'username', 'Gender']].groupby(['f_
dopa_group', 'username']).mean().reset_index()
df_F_DOPA_gen.groupby(['f_dopa_group', 'Gender']).size().reset_index(name='Cou
nt').set_index(['f_dopa_group', 'Gender'])
```

Out[50]:

		Count
**f_dopa_group**	**Gender**	
**Negative F-DOPA**	**0**	2
	**1**	4
**Positive F-DOPA**	**0**	15
	**1**	11

In [53]:
```
df_F_DOPA_age_MMSE = df_F_DOPA[['f_dopa_group', 'Age', 'mmse']].groupby(['f_do
pa_group']).describe()
df_F_DOPA_age_MMSE
```

Out[53]:

	Age								mmse		
	count	mean	std	min	25%	50%	75%	max	count	mean	st
**f_dopa_group**											
**Negative F-DOPA**	5930.0	66.120067	8.806272	51.0	58.0	72.0	74.0	75.0	5930.0	28.822260	1.[illegible]
**Positive F-DOPA**	25602.0	64.358409	12.066751	36.0	53.0	65.0	75.0	80.0	25602.0	29.621944	0.[illegible]

Healthy Controls

In [55]:
```
df_controls = df_controls[['group', 'mmse_group', 'username', 'Age', 'Gender',
'mmse']]

# filter to the healthy group:

df_controls = df_controls[df_controls['mmse_group']=='Healthy']
print('Number of healthy subjects in full sample:')
print(len(df_controls['username'].unique()))
print('Need to choose: 13 males (0) and 7 females (1), with mean age 65 and me
an mmse 29')
```

```
Number of healthy subjects in full sample:
40
Need to choose: 13 males (0) and 7 females (1), with mean age 65 and mean mms
e 29
```

In [58]:
```
df_controls_gen = df_controls[['mmse_group', 'username', 'Gender']].groupby
(['mmse_group', 'username']).mean().reset_index()
df_controls_gen.groupby(['mmse_group', 'Gender']).size().reset_index(name='Cou
nt').set_index(['mmse_group', 'Gender'])
```

Out[58]:

		Count
**mmse_group**	**Gender**	
**Healthy**	**0**	18
	**1**	22

In [60]:
```
df_controls_age_mmse = df_controls[['mmse_group', 'Age', 'mmse']].groupby(['mm
se_group']).describe()[[('Age', 'mean'), ('Age', 'std'), ('mmse', 'mean'), ('m
mse', 'std')]]
df_controls_age_mmse
```

Out[60]:

	**Age**		**mmse**	
	**mean**	**std**	**mean**	**std**
**mmse_group**				
**Healthy**	72.232713	9.636405	29.03749	0.808459

randomly select comparable participants from the control group

In [61]:
```
df_controls['mmse_group'].unique()
```

Out[61]:
```
array(['Healthy'], dtype=object)
```

In [63]:
```
# randomly choose 7 females:

df_controls_f = df_controls[df_controls['Gender']==1][df_controls['Age']<=78].
groupby(['username', 'Gender']).mean().reset_index()
df_controls_f = df_controls_f.sample(n = 7)
df_controls_f[['Gender','Age','mmse']]
```

```
/opt/tljh/user/lib/python3.6/site-packages/ipykernel_launcher.py:3: UserWarni
ng: Boolean Series key will be reindexed to match DataFrame index.
  This is separate from the ipykernel package so we can avoid doing imports u
ntil
```

Out[63]:

	Gender	Age	mmse
**1**	1	61	28
**0**	1	59	28
**3**	1	77	29
**6**	1	66	30
**10**	1	62	29
**7**	1	77	29
**2**	1	70	30

In [64]:

```
# randomly choose 13 males:

df_controls_m = df_controls[df_controls['Gender']==0][df_controls['Age']<=74].
groupby(['username', 'Gender']).mean().reset_index()
df_controls_m = df_controls_m.sample(n = 13)
df_controls_m[['Gender','Age','mmse' ]]
```

```
/opt/tljh/user/lib/python3.6/site-packages/ipykernel_launcher.py:3: UserWarni
ng: Boolean Series key will be reindexed to match DataFrame index.
  This is separate from the ipykernel package so we can avoid doing imports u
ntil
```

Out[64]:

	Gender	Age	mmse
**4**	0	74	28
**5**	0	68	30
**9**	0	56	28
**7**	0	70	29
**10**	0	67	30
**3**	0	66	30
**11**	0	66	30
**6**	0	61	29
**13**	0	71	29
**0**	0	70	28
**8**	0	66	30
**12**	0	71	28
**2**	0	66	28

In [73]:
```
df_comparable_controls = df_controls[df_controls['username'].isin(usernames)]
df_comparable_controls_users = df_comparable_controls.groupby(['username']).me
an().reset_index()
df_comparable_controls_users[['Age','Gender','mmse']]
```

Out[73]:

	Age	Gender	mmse
**0**	59	1	28
**1**	70	1	30
**2**	77	1	29
**3**	56	0	30
**4**	63	1	30
**5**	66	0	28
**6**	66	0	30
**7**	74	0	28
**8**	68	0	30
**9**	61	0	29
**10**	70	1	29
**11**	70	0	29
**12**	66	0	30
**13**	62	1	29
**14**	66	1	29
**15**	56	0	28
**16**	67	0	30
**17**	66	0	30
**18**	71	0	28
**19**	71	0	29

In [74]:
```
len(df_comparable_controls_users['username'].unique())
```

Out[74]: 20

In [79]:
```
df_comparable_controls.groupby(['f_dopa_group']).describe()
```

Out[79]:

	Age								Gender			
	count	mean	std	min	25%	50%	75%	max	count	mean	...	[illegible]
**f_dopa_group**												
**Negative F-DOPA**	29533.0	66.611215	5.331983	56.0	63.0	66.0	70.0	77.0	29533.0	0.378323	...	

1 rows × 24 columns

In [80]:
```
df_final_age_mmse_controls = df_comparable_controls[['mmse_group', 'Age', 'mmse']].groupby(['mmse_group']).describe()[[('Age', 'mean'), ('Age', 'std'), ('mmse', 'mean'), ('mmse', 'std')]]
```

all groups

gender

In [84]:
```
df_gen = df_demo_final[['f_dopa_group', 'username', 'Gender']].groupby(['f_dopa_group' ,'username']).mean().reset_index()
df_gen.groupby(['f_dopa_group', 'Gender']).size().reset_index(name='Count').set_index(['f_dopa_group', 'Gender'])
```

Out[84]:

		Count
**f_dopa_group**	**Gender**	
**Negative F-DOPA**	**0**	15
	**1**	11
**Positive F-DOPA**	**0**	15
	**1**	11

age and mmse

In [85]:
```
df_demo_final[['f_dopa_group', 'Age', 'mmse']].groupby(['f_dopa_group']).describe()[[('Age', 'mean'), ('Age', 'std'), ('mmse', 'mean'), ('mmse', 'std')]]
```

Out[85]:

	Age		mmse	
	**mean**	**std**	**mean**	**std**
**f_dopa_group**				
**Negative F-DOPA**	66.529087	6.056023	29.049798	0.844488
**Positive F-DOPA**	64.358409	12.066751	29.621944	0.553874

In [86]:
```
# healthy group:

df_demo_final[df_demo_final['f_dopa_group']=='Negative F-DOPA'].groupby(['Gender']).describe()[[('Age', 'mean'), ('Age', 'std')]]
```

Out[86]:

	Age	
	mean	std
Gender		
0	67.265534	5.404352
1	65.536414	6.710311

In [87]:
```
df_demo_final[df_demo_final['f_dopa_group']=='Negative F-DOPA'].groupby(['Gender']).describe()[[('mmse', 'mean'), ('mmse', 'std')]]
```

Out[87]:

	mmse	
	mean	std
Gender		
0	29.003635	0.850378
1	29.112023	0.832469

In [88]:
```
# pd group:

df_demo_final[df_demo_final['f_dopa_group']=='Positive F-DOPA'].groupby(['Gender']).describe()[[('Age', 'mean'), ('Age', 'std')]]
```

Out[88]:

	Age	
	mean	std
Gender		
0	63.541729	11.237702
1	65.470572	13.030196

In [89]:
```
df_demo_final[df_demo_final['f_dopa_group']=='Positive F-DOPA'].groupby(['Gender']).describe()[[('mmse', 'mean'), ('mmse', 'std')]]
```

Out[89]:

	mmse	
	mean	std
Gender		
0	29.685273	0.583039
1	29.535701	0.498747
